# Factors influencing the use of obstetric care during childbirth in rural Sudan: a qualitative study among healthcare providers

**DOI:** 10.12688/gatesopenres.16373.1

**Published:** 2025-12-16

**Authors:** Sophie Kellner, Raquel González, Cristina Enguita-Fernàndez

**Affiliations:** 1 Bernhard Nocht Institute for Tropical Medicine, Hamburg, 20359, Germany; 2 Universitat de Barcelona (UB), Barcelona, Catalonia, 08036, Spain; 3 ISGlobal, Barcelona, 08036, Spain

**Keywords:** Barriers, access to maternal healthcare, healthcare utilization, Sub-Saharan Africa, Sudan

## Abstract

**Background:**

It is estimated that over 287,000 women die annually from childbirth complications, with a substantial proportion living in sub-Saharan Africa. Access to professional obstetric care is crucial for averting these deaths, making it a global health priority. Urgency is heightened by limited literature regarding access to obstetric care, especially in Sudan’s rural areas, where maternal mortality peaks. Therefore, the study objective was to identify the perceived barriers and facilitators of accessing obstetric services during childbirth in rural Sudan, specifically in Nuba Mountains, a conflict-ridden isolated region, from the perspective of health experts.

**Methods:**

An exploratory qualitative study was conducted from March to May 2023, involving remote in-depth interviews and online questionnaires with 11 health professionals working in maternal and reproductive health in rural Sudan. Thematic analysis was used for the data analysis.

**Results:**

Challenges in accessing professional obstetric care in rural Sudan are influenced by sociocultural factors, healthcare perceptions, surrounding individuals, and structural factors. The main barriers identified include social factors such as widespread acceptance of home birth, gender inequality, and family influence; structural barriers like long distances, lack of transport, a deficient referral system, and reliance on untrained healthcare workers; and individual-level factors including low health awareness and limited decision-making autonomy. These barriers are further compounded by political instability, conflict, and displacement in the Nuba Mountains, which severely constrains women’s access to obstetric services.

**Conclusion:**

While many barriers align with existing literature, unique challenges in Nuba include centralized care, high acceptance of death, lack of a functioning communication network, and an unstable political situation.

Barriers preventing women from seeking professional obstetric care during childbirth are complex, with particular challenges in Nuba due to its remoteness and political instability. Future assessments and improvements in care in Sudan should incorporate women’s perspectives to ensure a more comprehensive and multidimensional approach.

## Introduction

Over the past two decades, approximately 10.7 million women worldwide have died from obstetric complications such as hemorrhage, hypertensive disorders, and sepsis. ^1, 2^ More than two-thirds of these deaths occurred in sub-Saharan Africa, and almost all of these deaths could have been prevented. Sub-Saharan Africa has the highest maternal mortality rate, with 533 maternal deaths per 100,000 live births. The risk of a woman dying during childbirth has declined -significantly worldwide, but inequality between and within countries remains. The bordering countries of Sudan show these differences. While Egypt, with 17 maternal deaths per 100,000 live births, is one of the countries with the lowest maternal mortality ratio in Africa, South Sudan, with 1223 per 100,000, is among the countries with the highest ratio. ^2^ Sudan’s maternal mortality ratio for 2020 was 270 per 100,000 live births and the neonatal mortality rate in 2021 was 26.7 deaths per 1,000 live births, ^2, 3^ which makes Sudan one of the countries in Sub-Saharan Africa where childbirth continues to be associated with a high risk for both the mother and child. ^2^ Access to quality obstetric care remains a challenge for many women worldwide, especially those in rural areas, leading to severe consequences such as maternal or neonatal death. ^4, 5^ The reasons why women do not seek professional obstetric care are manifold and difficult to penetrate. ^4, 5^

Previous studies on barriers to skilled obstetric care in low-middle-income countries in Sub-Saharan Africa and Southeast Asia revealed diverse challenges, including socioeconomic factors, high service costs, healthcare system inefficiencies, and sociocultural influences. ^5, 7^ Reviews of sub-Saharan African countries such as South Sudan and Ethiopia echoed similar themes, emphasizing issues such as geographic accessibility, service availability, transportation constraints, cultural influences, family support, economic factors, and healthcare quality. ^8– 10^ Despite public health measures, these interconnected barriers persist, indicating reliance on untrained birth attendants, poorly equipped clinics, and cultural, infrastructural, and economic influences on decision making. Especially in conflict and post-conflict settings across sub-Saharan Africa maternal health services are often severely disrupted. Studies from Burkina Faso and Ethiopia report that insecurity and armed conflict have led to significant declines in service utilization and worsened maternal outcomes. ^30, 31^ Evidence from other conflict-affected African countries underscores how fragile health systems, combined with displacement and insecurity, exacerbate barriers to accessing care. ^32^ Taken together, these findings point to the ongoing substantial investments in women’s health to address challenges in obstetric care access and utilization, particularly in healthcare infrastructure, workforce development, community-level health education, and policy initiatives that improve accessibility, availability, affordability, and acceptability of care. ^5, 7– 10^

Sudan’s health system is characterized by chronic underinvestment, political instability, and strong regional disparities, resulting in limited and unequal access to care. ^33^ Only 70% of the population can access a health facility within 30 minutes, and 80% within one hour. However, the quality of care remains inadequate. Merely half of facility visitors are seen by skilled health professionals, contributing to some of the region’s lowest maternal and child health service coverage. Maternal mortality is primarily driven by the absence of skilled birth attendants and limited access to antenatal and emergency obstetric care. While 85% of pregnant women attend at least one antenatal visit, just over 50% complete the recommended four visits. Furthermore, only 34% of young mothers and their newborns receive postnatal care. ^34^

Although Sudan’s maternal mortality ratio indicates that Sudan is not one of the countries where women’s healthcare is most critical, ^2^ there are wide disparities in healthcare services within the country. Due to the uneven distribution of healthcare resources, there are significant regional disparities in healthcare delivery and access, especially in the rural and conflict-affected areas. ^11^ These areas continue to face critical shortages in skilled birth attendance and emergency obstetric services. ^33^ Given these disparities within Sudan, maternal mortality ratio needs to be contextualized by encouraging a regional perspective. While previous research suggests that women in rural Sudan face challenges in accessing professional obstetric services, ^5, 7, 10^ there is a scarcity of studies employing qualitative methodologies that can generate more contextualized data and ensure a deeper understanding of these challenges. This emphasizes the need for further research on the utilization of obstetric care in rural areas of sub-Saharan Africa, particularly in Sudan’s rural regions, where maternal death incidences peak.

Across sub-Saharan Africa, socio-cultural norms play a decisive role in shaping maternal health-seeking behaviors. Reviews consistently identify gendered decision-making, stigma around family planning, and reliance on traditional birth attendants as factors that limit women’s engagement with formal obstetric services. ^8^

Therefore, the objective of this qualitative study was to identify the perceived barriers and facilitators of accessing obstetric services during childbirth in rural Sudan from the perspective of professionals working in the field of maternal and reproductive health. Specifically, the study sought to explore how structural and sociocultural factors could affect pregnant women’s access to obstetric services and assess the influence of individuals in their immediate surroundings on healthcare decisions during childbirth.

## Methods

### Study design

An exploratory qualitative study was conducted, following the principles of Grounded Theory, which is an inductive approach, whereby no theoretical framework was used to guide the research. ^15^ Primary data were collected from March 2023 to May 2023, through semi-structured, in-depth expert interviews, conducted remotely, and self-completed interviews through online questionnaires with healthcare providers to explore their perspectives on barriers and facilitators to obstetric services during childbirth in rural Sudan. Prior to data collection, a literature review was conducted to gather existing knowledge relevant to the study objective and geographic area.

### Study setting

In the region of Nuba Mountains, located within South Kordofan state, a prolonged armed conflict has unfolded between Sudanese government forces and the non-state armed group Sudan People’s Liberation Movement-North (SPLM-N) over several decades. ^12^ South Kordofan, bordering South Sudan, is partially inaccessible due to the conflict, posing significant health risks for its nearly 2 million residents. ^13, 14^ The SPLM-N insists on the delivery of humanitarian aid across the border with South Sudan, while the Government promotes aid from within Sudan. As a result, humanitarian access to these areas remains inconsistent, and there is virtually no information about the current humanitarian and health situation. The area is difficult to access and is therefore not included in regular data collection activities coordinated by the United Nations or the Government of Sudan. ^12, 14, 28^ This dynamic has led to variations in humanitarian access and a deficiency in essential services such as healthcare and education. ^12– 14^ The humanitarian challenges triggered by years of governmental neglect in Sudan include displacement, critical food insecurity, and infrastructural damage. For the communities in the affected regions, access to essential social services is either severely limited or entirely absent. ^26^ In South Kordofan, entrenched gender roles and traditional social norms play a significant role in perpetuating harmful practices, including child marriage, type-III female genital mutilation (FGM), and Adal circumcision—the re-stitching of genital tissue after childbirth. ^27^ Within this context, women encounter challenges accessing contraception, antenatal care, and obstetric services, ^12^ with a significant number giving birth at home without assistance. ^14^ Women facing complications during childbirth are often forced to undertake perilous, days-long journeys—sometimes even crossing active conflict zones—in order to reach emergency obstetric care. ^12^ According to a needs assessment in Nuba, 91% of the women gave birth at home and 30% of them unassisted. ^23^ In the study from Kassala, in Sudan however, 56.8% of women had skilled birth attendants and 66.3% gave birth at home. ^6^

Currently, the SPLM-N controls various areas in South Kordofan, with the Nuba Mountains predominantly situated within these monitored regions. Presently, the region remains in a transitional phase without a peace agreement with the government, making the health situation in the Nuba Mountains (Nuba) distinct from the rest of the state, necessitating separate consideration. ^14^

Since April 2023, the renewed conflict in Sudan has had substantial consequences for civilians, affecting nearly half of the population. Numerous individuals have lost their lives, requiring humanitarian aid or facing displacement. ^16^ During preparation and execution of this study, the extent of this conflict and its impact on the humanitarian situation was not fully evident, and thus, not considered.

### Participants and sampling methods

The participants were experts working in maternal and reproductive health in Nuba and included local hospital staff, such as midwives, physicians, and nurses. In addition, physicians and nurses of non-governmental organizations (NGOs) working in the area, who have insights into the topic, were included. The identification of respondents and the recruitment process were non-probabilistic and were based on a combination of reasoned choice (purposive selection) and convenience sampling. Snowball sampling was used as a complementary strategy. The invited participants, who were willing to participate and provided informed consent, were included in the study. The sample size was determined by the saturation point, whereby all themes were exhaustively explored, and no new themes emerged in subsequent interviews.

### Data collection

The data were collected through in-depth interviews and online questionnaires. A semi-structured interview guide was developed by the research team specifically for this study based on existing literature and expert knowledge in the field of maternal and reproductive health in rural Sudan. The questionnaire included both open-ended and close-ended questions designed to gain insight into participants’ perspectives on women’s practices related to obstetric care and childbirth in Nuba.

In-depth interviews were conducted over the phone in German with employees of a German-speaking aid organization active in the region and in English with participants from Sudan or other countries. Interviewees were asked to choose a private setting for confidentiality. Interviews, lasting between 35-55 minutes, were digitally recorded, and notes were taken.

Additionally, an adapted online questionnaire was employed, specifically designed for participants with difficult access to electronic devices or stable internet connections. It mirrored the interview guide, featuring similar open-ended questions for written responses, alongside some close-ended questions for selection. Overall, this approach ensured that the quality and depth of responses did not substantially differ between the interviews and the online questionnaires. An online form developed using Google Forms was sent via email or other online platforms. Participants’ responses were automatically collected in a Google spreadsheet.

### Data management and analysis

The collected data were digitized, and the audio files were transcribed verbatim, if in English, or translated into English. The transcriptions and translations were subjected to quality control and compared with the audio files to verify accuracy.

The interview transcripts, field notes, and digitized data from the online questionnaires were manually coded using Microsoft Excel. Data were analyzed using a thematic analysis approach, which consisted of identifying emerging themes relevant to the study aim. A general outline of themes and codes was developed, which was updated as themes emerged. Data analysis followed a cyclical process of data collection and preliminary analysis and culminated in the final analysis after completing data collection. This study adheres to the reporting standards outlined in the SRQR guidelines for reporting qualitative studies ^19^ (Additional File 1).

### Ethics, informed consent procedures, and confidentiality

The study was conducted in accordance with the Good Clinical Practice Guidelines, the provisions of the Declaration of Helsinki, and local rules and regulations.

Ethical approval for the study was granted by the Ethics Review Committee of the Hospital Clínic de Barcelona, Spain (CEIC) [Reg. HCB/2023/0107] on the 02/03/2023.

As the study was conducted entirely online with participants from multiple countries and without involvement of local institutions, additional ethical approvals were not required. ^29^

Participants provided written or oral consent for the interviews and audio recordings. Given that the interviews were conducted remotely, verbal consent was recorded before the interview began. ^18^ All names in the transcripts were deleted to guarantee anonymity.

## Results

### Participants’ characteristics

Eleven health experts were interviewed (seven in-depth interviews and four online questionnaires) between March and May 2023. Sociodemographic characteristics of the participants are presented in the following Table 1.

**Table 1.** Characteristics of respondents.

	Participants (n = 11)
Characteristics	n
**Gender**	
Male	5
Female	6
**Age (years)**	
25-35	5
36-45	3
46-55	1
56-65	2
**Nationality**	
Sudanese	4
German	5
Others	2
**Occupation**	
Physician	5
Nurse	2
Midwife	1
Traditional Birth Attendant	1
Clinical Officer	1
Nutritionist	1
**Marital status**	
Married	5
Single	6
**Religious affiliations**	
Christian	8
Non-religious	3
**Time of working in Nuba**	
<1 year	2
1-5 years	5
6-10 years	2
>10 years	2

### Context characteristics

While offering nuanced insights into healthcare and maternal health-seeking practices based on their own views and experiences, the interviewees also provided a rich and detailed description of the context.

*War history and political situation*

The participants portray Nuba as an unstable region. Many years of war, conflict, and displacement have left the people in the region isolated. As there is still no peace, the region remains cut off and abandoned by the Government of Sudan. Humanitarian aid must therefore be provided across South Sudan’s border. However, the crossing is fraught with security issues and enormous delays often occur. Some participants described the political situation as hopeless and assumed that the health situation of the population would only improve with a peace agreement.

*Positive trends in regional development*

Many participants mentioned a general positive trend in the development of the region. In recent years, there has been some change for the better, for example, enhanced health education through increased NGO presence and a general rise in formal education driven by more children attending school.

*“Those [pregnant women] who come for the checkups […] is rather the new generation. They are first or second-time mothers who are still young, who have an education, and who want their child to be born healthy. They see the sense in the fact that a preventive examination can have an influence on the birth.”* (Interview 3, male)

*Healthcare system and practices*

According to the participants, there were 200-220 health facilities at different levels in Nuba, of which an estimated 70% were supported by NGOs. Nevertheless, most health facilities are poorly equipped, especially in the less centrally located areas. Participants explained that healthcare is still very rudimentary; in some areas even non-existent.

There are two major referral hospitals where surgical procedures can be performed. The two hospitals are based in the centrally located county of Heiban and are an hour’s drive from each other. These hospitals are the only ones in the entire region, with an estimated population of 2 million, where caesarean section and other surgeries can be performed.

According to hospital staff interviewed, there are an estimated 28 trained midwives, but it is unclear if or where they are practicing.

As stated by the participants, women were hardly relieved of their domestic duties during pregnancy. During this time, they must perform hard physical labor. As a result, some interviewees described repeated miscarriages or premature labor resulting from hard physical labor during pregnancy.

Based on participants’ responses, some women give birth alone or birth is performed by an untrained person, such as an older woman of the family, a village elder woman, or a traditional birth attendant (TBA). Often, even a TBA is called in only when complications during childbirth arise, often when it is already too late and irreversible complications have occurred. For home births, families and TBAs are often not equipped with sterile materials or emergency medication.

*“There are some [pregnant women] who may just deliver alone. Maybe unless there is a complication, that’s when they run and call the TBA.”* (Interview 2, female)

Another special characteristic in Nuba is the so-called co-patients. This locally used term refers to family members, friends, or neighbors who accompany patients to the hospital or health facility and take over the entire care of the patients. Bringing a co-patient during a hospital stay is essential.

*“They need someone […] who does all the nursing care, who does the laundry, who cooks, and who cares for the patient. That is not done by the staff or cannot be done at all.”* (Interview 4, female)

### Factors influencing utilization of obstetric services

**Figure 1.**
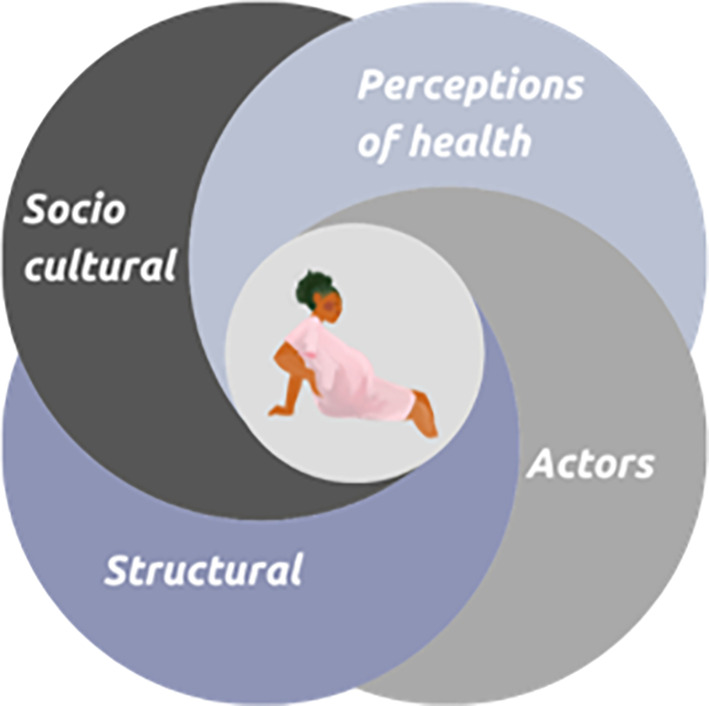
Main factors influencing the use of obstetric care.

Four overarching themes and 17 factors were identified in the interviews. These four themes are shown in the Figure 1 below.

*① Sociocultural*

*Gender roles*

Participants universally highlighted a critical issue: the lack of women’s empowerment and gender-based imbalances. This issue was underscored by the lack of assertiveness among women who often refrained from contradicting their husbands or family elders. As mentioned by the participants women’s opinions were consistently undervalued and they faced unequal participation in decision-making, notably in health matters such as family planning and professional obstetric care choices. Furthermore, the broader neglect of women’s roles in the healthcare system, encompassing inadequate funding, financial investment in health, and political decisions, has emerged as a notable concern.

*“For the women in Nuba, it is the tragedy that they have to provide for the whole family and work the hardest - even when they are pregnant. And there is hardly any relief or no legislation that relieves them […] for the fact that they keep this whole country running, they are not appreciated at all. Regarding the numbers on Domestic Violence, Gender Based Violence, women’s rights: it’s pretty dark.”* (Interview 3, male)

As indicated by the participants, some maternal health-related issues, such as abortion or family planning, were highly stigmatized in the community and are often considered taboo. This stigma could discourage women from engaging with health facilities, as fear of condemnation or rejection can lead to avoidance of institutional care, including childbirth.

*Lack of formal education & low health awareness*

All participants mentioned a low level of school education as well as low knowledge about health. This refers to pregnant women and the people surrounding them in their daily lives, as well as the people who make medical decisions for pregnant women, such as older women in the family, husbands, or TBAs.

As stated by the participants, illiteracy or a low education level is also associated with low health awareness, which refers to a lack of knowledge, understanding, or concern about one’s own physical or mental condition, as well as that of the unborn child. Through poor health literacy, pregnant women and those surrounding them are not aware of the benefits of giving birth with professional healthcare providers or visiting regular preventive health measures, such as antenatal care. Furthermore, it leads to the fact that danger signs during childbirth are not recognized and that professional medical help is not used or is used too late.

*“Most pregnant women in Nuba are not aware of the benefits of delivering in the hospital because of high illiteracy rate in Nuba. Majority of women have never gone to school- So it is sometimes difficult to explain, that coming earlier to hospital for delivery would save both lives”* (Questionnaire 2, female)

*Perceptions around death*

According to interviews, how the death of a child or a woman during childbirth is viewed and accepted also influences whether professional services during childbirth are used.

Many participants explained that death is something ubiquitous and normal in Nuba and is often accepted as God’s will. Many families have already experienced child loss, which diminishes the surprise and contributes to a high level of acceptance when the mother or child dies in childbirth.

*“In general, death is much more ubiquitous and omnipresent in Nuba and thus more normal […] everyone knows such stories and many have already had miscarriages, and many have already lost children. It is not totally surprising and shocking for people most of the time. It never comes completely unexpected when a child or the mother dies.”* (Interview 1, female)

*Tradition and cultural norms*

In accordance with the interviewees, one of the main reasons why women do not use obstetric care is the widely accepted social norm that birth is something common and therefore no professional help is needed. Phrases such as *“because that’s the way it has always been”* (Interview 1, female) came up frequently in the interviews.

*② Perceptions of health and healthcare*

*Low risk perception of childbirth*

As per the interviewees, the benefits of institutional childbirth were mostly unknown. Birth is usually perceived as a natural and uncomplicated event in Nuba. The potential for risks and the possible need for medical care during childbirth are usually underestimated.

*“The issue and the problem during delivery is not something the hospital or modern medicine can solve- it is a local problem.”* (Interview 5, male)

*Previous contact with the healthcare system*

Participants mentioned that prior contact with the healthcare system during pregnancy could positively influence seeking obstetric care during birth. This contact may occur through antenatal care, in which women are encouraged to give birth at healthcare facilities. Satisfaction with professional care in previous pregnancies may encourage women to seek similar care in their next pregnancy.

*Lack of priorities*

As stated by the participants, it is a question of prioritizing whether healthcare is used or not. Many women have obligations at home, for example, to take care of other children or to work in the field. This work would otherwise be neglected, and there is usually no one who could continue this work.

*Perceived quality of care*

The listed facilitators included the construction of new spaces for maternity units and the distribution of non-food items as an incentive to visit a health facility. Another factor mentioned was the increased likelihood of women seeking hospital care for childbirth when there was awareness of the presence of international professionals in the locality, such as obstetricians from international NGOs.

The participants also identified expectations of “poor quality of care” as a barrier, including concerns about uncomfortable or substandard facilities, anticipation of inadequately qualified staff, and expectations of poorly equipped facilities with medication shortages. Fear and skepticism about hospital visits stemmed from the belief that people are more likely to die in hospitals, often due to delayed arrivals. Anxiety regarding surgical interventions, particularly caesarean section, and privacy concerns during the birthing process were also barriers mentioned. Additionally, there was a lack of trust in modern medicine, and discomfort with potentially inexperienced or male staff members.

*③ Actors*

*Influence of family and peers on decision-making*

It became clear during the interviews that the decision on whether to give birth in a hospital was very rarely made by the pregnant woman herself. Usually, this decision is made by the head of the family, usually either the husband or an older woman in the family. Participants described family influence as a major barrier to skilled obstetric care.

*“When it comes to health-seeking behaviour, the woman doesn’t have the real authority to decide. The mandate has to come from the husband or elder members of the family. They have to get consent even on important things. [...] So if the husband is ignorant about the needs for our [healthcare workers] help, he may make a decision that will hinder her going.”* (Interview 2, female)

*Alternative traditional health providers*

Inadequately trained traditional health providers, such as TBAs and traditional healers, also have a great influence on the decision-making process. According to the interviewees, although some of them are experienced in obstetrics, TBAs in Nuba usually have little or no professional training and often do not recognize signs that a birth does not progress well until severe complications appear.

*“By the time they [untrained people conducting childbirth] are deciding for referral, it will be too late getting a child dying or a mother already dying because of a delay in transport. On their way reaching to the hospital, it is already complicated. The mother is in bad condition because of the delay of a TBAs who has not enough knowledge on how and when to refer a mother.”* (Interview 6, female)

*④ Structural factors*

*Costs and economic situation of pregnant women and their families*

High costs (direct and indirect) coupled with the low economic status of the population were cited as barriers by the participants.

Based on the interviews, in most hospitals and health facilities, women did not have to pay money or only a nominal fee for obstetric care. Participants indicated that the nominal fees are not a barrier to obstetric care because they are usually affordable. It would only be considered a barrier due to the misconception that giving birth in a hospital is expensive, and therefore, women might prefer home birth.

Indirect costs include food and laundry during the stay in the health facility and neglected responsibilities at home, such as fieldwork and caring for children. The same applies to the co-patient. Neglected domestic duties or professional activities for the patient and her companion have financial disadvantages that are described as barriers. Another barrier is the high transport costs.

*“They [pregnant women] have a co-patient that need to be at home to be farming or taking care of other children. So there are kind of human costs […].”* (Interview 5, male)

*Geographical and infrastructural conditions*

Nuba is a very rural and remote area, which is why the distances are often long and many smaller villages are isolated.

*“You hear that again and again when you go to the remote areas. For those [pregnant women] who don’t have access to midwives or healthcare, the woman would be put on a bed and then carried to the next road - and then hope that a car will stop there or that it will pass by in the next few days. But then, these women regularly die on the way to the hospital or have intrauterine fetal death.”* (Interview 3, male)

Participants indicated that the travel time to reach the nearest health facility was very long, usually more than two hours, sometimes even several days. Poorly constructed roads have also been described as significant problems.

As maintained by the interviews, it was difficult to access care due to the weather and environmental conditions. In particular, the rainy season poses major difficulties, for example, impassable roads and, as a result, being cut off.

*“[...] 20 minutes after she [a young pregnant women] arrived, she died [...] it made me very sad that this woman had to come to us after all those hours. She came from a distant village. And because of the rain, they [the women and her family] couldn’t set off directly. It took a while until they were able to come. At the moment she reached us, the body was no longer able to cope with it. Then, after everything she had gone through, the moment she reached us, she died.* (Interview 4, female).

Another problem described by all participants was the lack of transportation to health facilities. All participants stated that most women had to walk to get there, even if they were already in labor. Participants describe the possibility of getting to the hospital by a means other than walking as a “ *matter of luck*” (Interviews 1, 2), for example, if a passing car offers them a ride.

Another major problem that leads to delayed or non-utilization of qualified obstetric care is the absence of a functioning referral system. Families have hardly any possibility of reaching the hospital quickly in the case of obstetric complications. There are no standby ambulances or other public systems to assist with referrals. Furthermore, there are hardly any network or communication possibilities, for example, to exchange patient information, announce a transfer or referral, or ask for help.

A general shortage of health facilities and hospitals has also been frequently described. Centralized care in terms of geographical accessibility and distribution of care services presents a significant healthcare challenge in Nuba. With both referral hospitals located in the itself rural central region, the concentration of essential services in one area leads to the neglect of distant communities, depriving them of access to critical medical procedures.

*Quality of healthcare services*

All participants described a deficient infrastructure of the health system, including a lack of medical equipment, devices, and medicines. Almost all health facilities rely on external aid and are supported by NGOs operating in the region. Due to the political situation, humanitarian goods must be delivered via countries other than Sudan. Frequent floods interrupt the supply chain and cause delays.

One barrier to the use of obstetric care for childbirth is poorly trained healthcare workers. Many employees working in the region do not have professional training. Typically, they are trained by existing staff in the hospital or undergo short, general training, often for only a few months. Consequently, complications during the birthing process are often detected too late.

A summary of the themes, factors, and individual barriers and facilitators is listed below ( Table 2).

**Table 2.** Themes, factors, and description of barriers and facilitators to obstetric care.

Main theme	Factors	Description of factors
Sociocultural	Gender roles	Gender inequality
		Stigma
	Education & health awareness	Lack of education of PW and family
		Low health awareness of PW and family
	Perceptions around death	High acceptance of death
		Influence on acceptance of death
	Tradition & cultural norms	Home birth culture
		Elderly women/TBAs assist delivery
Perceptions of health & healthcare	Low risk perception of childbirth	Underestimated risk of childbirth
		Underestimated benefit of institutional childbirth
	Lack of priorities	Domestic obligations
	Perceived quality of care	Expectation of poor facilities
		Expectations of poorly equipped HF
		Expectation of unqualified staff
		Fear & scepticism
		Lack of trust
		Privacy concerns
Actors	Influence of family & peers on Decision-making	Decision-making: male head of family
		Decision-making: female head of family
		Decision-making: PW
		Family influence
		Peer influence
	Alternative traditional health provider	TBAs
		Traditional healer
Structural factors	Costs & economic situation	Direct costs
		Indirect costs
		Economic status
	Geographical & infrastructural conditions	Remoteness
		Long distances
		Meteorological conditions
		Means of transport
		Road conditions
		Referral system
		Scarce of HF
		Centralized care
		Network & communication
	Quality of healthcare services	Poorly equipped HF
		Lack of healthcare workers
		Poorly trained healthcare workers
		Provider-patient relationship

## Discussion

This study identified perceived barriers and facilitators, based on health experts’ perceptions on the use of professional obstetric care during childbirth in rural Sudan.

The findings indicate that challenges in accessing skilled obstetric care contribute to prevalent home births, with only a minimal number of women opting for skilled care providers.

The main factors hindering the use of obstetric care services, emphatically mentioned by almost all participants, were home birth as a cultural norm, low health awareness, and gender inequality. Many of the main factors were structural, including long travel times, lack of means of transport or referral systems, poor availability, lack of health facilities and centralized care, and poorly trained staff.

Gender inequality in Nuba significantly impedes women’s access to skilled obstetric care due to limited empowerment and involvement in decision-making. Additionally, low formal education and limited health awareness contributed to delayed or absent referrals. Both points align with the existing literature in various countries, including South Sudan, Ethiopia and Democratic Republic of Congo-countries sharing sociocultural, economic, and geographic traits with Nuba, but also a similar conflict-affected context. ^5, 8– 10, 22^

In Nuba, where death is normalized and widespread, there is high acceptance of mortality, even among young women and children. This acceptance could hinder obstetric care seeking, as prioritizing the woman’s or unborn child’s life may not be emphasized. While frequently highlighted in the interviews in this study, this aspect remains unaddressed in the literature, marking it as a notable barrier.

Consistent with findings from other conflict-affected countries such as South Sudan, Democratic Republic of Congo and Central African Republic, ^5, 7– 10^ respondents in our study emphasized family influence as well as the influence of TBAs as significant barriers to accessing skilled obstetric care. Moreover, as stated by the participants, in Nuba, TBAs are often not well trained and do not provide adequate assistance. Although evidence shows that training TBAs does not reduce maternal mortality, it can reduce perinatal and neonatal deaths. ^20^ There is a widespread lack of professional training among healthcare providers in Nuba. Systematic reviews show that emergency obstetric care training in LMICs in sub-Saharan Africa consistently enhances provider competence and clinical practice. Although robust evidence directly linking these training to improved maternal and neonatal survival is limited, emerging findings suggest potential reductions in neonatal complications, postpartum hemorrhage, and case fatality rates. Despite demonstrated improvements in knowledge, skills, and care quality, strong evidence of impact on patient outcomes remains insufficient. ^35, 36^ The interviews revealed the positive effect of previous contact with healthcare workers, which encouraged women to seek professional assistance. Similar positive effects of prior healthcare contact have been noted in studies conducted in South Sudan and Sudan. ^6, 10^

Perceived quality of care also plays a role in the decision-making process. While the construction of new maternity wards and the presence of international staff from NGOs were described as facilitators, expectations of uncomfortable facilities, poorly qualified staff, lack of equipment, fear, and lack of trust in modern medicine were described as barriers. Aspects that were also mentioned in the literature. ^5, 7, 10^

The study findings suggest an important impact of structural factors. This is broadly consistent with the literature, where distance, lack of transport, costs, poor referral practice, and poor roads were described as the main barriers. ^5, 7– 10, 22^ A systematic review emphasizes the critical nature of long travel times arising from the points mentioned, stating that the average time between the onset of a common major obstetric complication, postpartum hemorrhage, and death is two hours without medical intervention. ^5^ This illustrates the far-reaching effects that these delays can have.

In this study, high costs, especially the indirect costs of hospitalization, were identified as barriers. Here, the role of co-patients emerged as a distinct characteristic of the area, contributing to the characterization of Nuba as a depressed area while emphasizing the necessity for building supportive networks. However, it is intertwined with financial concerns for patients and the accompanying person, as both have to bear indirect costs. Participants thus described the presence of co-patients ambivalently: while they were seen as an essential source of support in the absence of sufficient healthcare staff, they also represented an additional financial and social burden for families. This ambivalence reflects both the strength of social support networks in Nuba and the financial strain they impose, highlighting the dual role of co-patients as facilitators and barriers to maternal care.

Along with this, all participants reported great difficulty arising from a lack of means of transport. Although a lack of referral practice and transportation is mentioned in the literature ^5, 7, 10, 22^ none of the studies indicated the absence of a functioning communication network, suggesting that an inadequate network may be a notable factor that further exacerbates the health situation of pregnant women in Nuba.

In addition, geographically centralized care and uneven health service distribution in Nuba pose a major healthcare problem, as studies show the frequent need for medical interventions, such as caesarean section, in case of labor complications. ^6^ The centralized care in Nuba seems to stand out, as this point has not been reported in other studies. These conditions cast doubt on the notion of home birth, shedding a different light on its feasibility and desirability. While home birth is deeply ingrained as a social practice, it may also persist as a sustained practice as a result of the obstacles women face in accessing health facilities.

Furthermore, logistical delays occur frequently due to the political situation and frequent floods during transport, which is consistent with the studies conducted in South Sudan. ^8– 10^ The fact that the delivery of humanitarian aid remains complicated makes the healthcare situation in Nuba compley and a political problem. This explicit complication from cross-border deliveries has not been mentioned in the literature and appears to be an explicit problem in Nuba.

A limitation of this study is the selection of health experts as interviewees, as they only revealed partial views on the topic, which were not based on the accounts of pregnant women. To address this constraint, we deliberately selected healthcare experts with extensive experience working in the region, many of whom have collaborated closely with pregnant women over several years. Nevertheless, we acknowledge that women’s direct voices would have further enriched the analysis. Since not all the respondents were from Sudan, the study may contain cultural bias, and the results may be influenced by personal values. Additionally, the exceptional living conditions of women in Nuba may not reflect the experiences of pregnant women in other rural areas of Sudan. However, a major strength of this study is that it is the first of its kind in Nuba and promotes a nuanced and contextualized view by focusing on a specific area in Sudan. These perspectives are necessary to avoid looking at health issues as a whole in a large country like Sudan.

Community-based public health awareness campaigns to promote safe childbirth targeting pregnant women, their relatives, and alternative healthcare providers should be pursued. Furthermore, training programs for healthcare workers should be established to ensure high-quality obstetric care. As there is often no alternative to home birth, ongoing training for TBAs would ensure that they can perform safe childbirth in the home setting. ^20, 37^

The findings highlight the necessity for a nuanced, multidimensional approach that considers the complex interplay of social, cultural, economic, and logistical factors in assessing and enhancing obstetric care utilization in the region. Therefore, a context-sensitive understanding of the perception of women’s perspectives on influencing factors is key to a comprehensive understanding of women’s health in Nuba.

## Conclusion

This study shows a large number of complex and multifactorial barriers that can prevent women from seeking professional help during childbirth.

However, the study suggested that the situation for pregnant women in Nuba is very specific compared to other areas of the country and points out some barriers to obstetric care that seem to be outstanding. These include strong centralized care, high acceptance of death among the population, the need for co-patients, and lack of functioning communication networks, as well as an unstable political situation.

Given the challenging context of Nuba, addressing these issues requires multiple intersecting approaches, considering the region’s distinct context, precarious political situation, and the exceptional remoteness of the region.

Building on the identified facilitators, future interventions should prioritize strengthening community awareness and engagement, supporting frontline health workers, and involving key decision-makers such as husbands, elder women, and traditional birth attendants to improve maternal health services. Moreover, future research should give greater priority to women’s own voices and perspectives, which are essential for developing context-sensitive and sustainable solutions.

## Consent & ethical approval statement

All participants provided informed consent prior to participating in the study. For remotely conducted interviews, verbal consent was obtained and recorded before the start of each interview. Participants were informed about the voluntary nature of the study and their right to withdraw at any time, and their responses were anonymized. Ethical approval for the study was granted by the Ethics Review Committee of the Hospital Clínic de Barcelona, Spain (CEIC) [Reg. HCB/2023/0107] on the 02/03/2023.

The study was conducted in accordance with the Good Clinical Practice Guidelines, the provisions of the Declaration of Helsinki, and local rules and regulations.

## Supplementary data

**Consolidated criteria for reporting qualitative studies (SRQR)**

**Table:** 

		Reporting item	Page number
**Title**			
	#1	Concise description of the nature and topic of the study identifying the study as qualitative or indicating the approach (e.g. ethnography, grounded theory) or data collection methods (e.g. interview, focus group) is recommended	1
**Abstract**			
	#2	Summary of the key elements of the study using the abstract format of the intended publication; typically includes background, purpose, methods, results and conclusions	1-2
**Introduction**			
Problem formulation	#3	Description and significance of the problem/phenomenon studied: review of relevant theory and empirical work; problem statement	2-3
Purpose or research question	#4	Purpose of the study and specific objectives or questions	3
**Methods**			
Qualitative approach and research paradigm	#5	Qualitative approach (e.g. ethnography, grounded theory, case study, phenomenolgy, narrative research) and guiding theory if appropriate; identifying the research paradigm (e.g. postpositivist, constructivist/interpretivist) is also recommended; rationale. The rationale should briefly discuss the justification for choosing that theory, approach, method or technique rather than other options available; the assumptions and limitations implicit in those choices and how those choices influence study conclusions and transferability. As appropriate the rationale for several items might be discussed together	3-4
Researcher characteristics and reflexivity	#6	Researchers' characteristics that may influence the research, including personal attributes, qualifications/experience, relationship with participants, assumptions and / or presuppositions; potential or actual interaction between researchers' characteristics and the research questions, approach, methods, results and/or transferability	N/A
Context	#7	Setting/site and salient contextual factors; rationale	4
Sampling strategy	#8	How and why research participants, documents, or events were selected; criteria for deciding when no further sampling was necessary (e.g. sampling saturation); rationale	4-5
Ethical issues pertaining to human subjects	#9	Documentation of approval by an appropriate ethics review board and participant consent, or explanation for lack thereof; other confidentiality and data security issues	6
Data collection methods	#10	Types of data collected; details of data collection procedures including (as appropriate) start and stop dates of data collection and analysis, iterative process, triangulation of sources/methods, and modification of procedures in response to evolving study findings; rationale	5
Data collection instruments and technologies	#11	Description of instruments (e.g. interview guides, questionnaires) and devices (e.g. audio recorders) used for data collection; if/how the instruments(s) changed over the course of the study	5-6
Units of study	#12	Number and relevant characteristics of participants, documents, or events included in the study; level of participation (could be reported in results)	4-5
Data processing	#13	Methods for processing data prior to and during analysis, including transcription, data entry, data management and security, verification of data integrity, data coding, and anonymization/deidentification of excerpts	5-6
Data analysis	#14	Process by which inferences, themes, etc. were identified and developed, including the researchers involved in data analysis; usually references a specific paradigm or approach; rationale	5-6
Techniques to enhance trustworthiness	#15	Techniques to enhance trustworthiness and credibility of data analysis (e.g. member checking, audit trail, triangulation); rationale	5
**Results/findings**			
Syntheses and interpretation	#16	Main findings (e.g. interpretations, inferences, and themes); might include development of a theory or model, or integration with prior research or theory	6-14
Links to empirical data	#17	Evidence (e.g. quotes, field notes, text excerpts, photographs) to substantiate analytic findings	6-14
**Discussion**			
Integration with prior work, implications, transferability and contribution(s) to the field	#18	Short summary of main findings; explanation of how findings and conclusions connect to, support, elaborate on, or challenge conclusions of earlier scholarship; discussion of scope of application/generalizability; identification of unique contributions(s) to scholarship in a discipline or field	14-17
Limitations	#19	Trustworthiness and limitations of findings	16
**Other**			
Conflicts of interest	#20	Potential sources of influence of perceived influence on study conduct and conclusions; how these were managed	21
Funding	#21	Sources of funding and other support; role of funders in data collection, interpretation and reporting	21

## Data Availability

The data supporting the findings of this study are available upon request from the corresponding author. The data were not publicly available because of information that could compromise the privacy of the research participants. To request access to the data, please contact Sophie Kellner ( Sophie_kellner@hotmail.de). Access will be granted to qualified researchers for non-commercial academic use, subject to approval of a data sharing agreement that ensures confidentiality and compliance with ethical guidelines. This study adhered to the “Standards for Reporting Qualitative Research” (SRQR) guidelines for reporting qualitative studies ^25^: (Supplementary data)
